# Social media, digital literacy, and career competence: a mixed methods study among university students in China

**DOI:** 10.3389/fpsyg.2025.1700213

**Published:** 2025-11-19

**Authors:** Chen Jian, Danting Zou, Nomahaza Mahadi

**Affiliations:** 1 School of Digital Economy and Trade, Guangzhou Maritime University, Guangzhou, China; 2 Azman Hashim International Business School (AHIBS), Universiti Teknologi Malaysia, Kuala Lumpur, Malaysia

**Keywords:** social media, digital literacy, career competence, Social Cognitive Career Theory, gamification, higher education, mixed methods

## Abstract

**Introduction:**

The widespread use of social media has transformed how university students develop knowledge and prepare for future careers. However, limited evidence exists on whether structured and purposeful engagement with these platforms can strengthen students’ digital literacy and career competence, particularly within the Asian higher education context.

**Methods:**

Guided by Social Cognitive Career Theory and gamified learning principles, this study employed a convergent parallel mixed-methods design. A one-month intervention was implemented with 44 undergraduates from three Chinese universities, involving structured social media tasks delivered through gaming and livestream activities.

**Results:**

Quantitative analyses revealed significant gains in digital literacy, professional skills, learning attitudes, and career competence. Digital literacy also emerged as a strong predictor of both professional skills and career competence. Complementary qualitative findings highlighted students’ enhanced self-regulation, stronger employability orientation, and a shift toward viewing social media as a professional rather than purely social tool.

**Discussion:**

These findings suggest that short, gamified social media interventions can effectively and sustainably enhance students’ digital capabilities and career readiness. The study extends the application of Social Cognitive Career Theory to media-rich learning environments and provides practical implications for higher education institutions seeking to strengthen graduate employability.

## 1 Introduction

In today’s rapidly evolving digital landscape, technology has become inseparable from university students’ academic and professional development. From a psychological perspective, such integration also influences key motivational and self-regulatory processes—central constructs in Social Cognitive Career Theory (SCCT)—that shape how students build employability skills and confidence.

Among these tools, social media platforms such as WeChat, TikTok, Xiaohongshu, Facebook, and LinkedIn have evolved from leisure spaces into dynamic environments for communication, self-regulated learning, and professional networking (Yang and Wang, 2021). Prior studies have documented this transition: Zachos et al. (2018) reviewed the educational uses of social media, Hamadi et al. (2022) examined its cooperative learning potential, and Ramzan et al. (2023) highlighted its motivational effects. Yet, from a psychological perspective, little is known about how such platforms shape students’ self-efficacy and outcome expectations—key constructs in SCCT—that drive digital literacy and career competence.

Despite these insights, the educational and professional potential of social media remains underexplored in Asian higher education (Foster et al., 2024). Much of the existing scholarship is Western-centric, leaving limited understanding of how structured, goal-directed social media use may influence digital literacy and career competence in Chinese and broader Asian contexts. This gap matters because the ability to leverage social media for knowledge acquisition, professional visibility, and opportunity discovery is increasingly a core dimension of graduate employability. However, previous studies, such as Mtawa et al. (2021), have rarely examined how structured, goal-directed social media activities can foster employability-related competences—such as self-regulated learning, digital problem-solving, and career planning—within non-Western higher education systems.

Digital literacy—defined as the ability to access, evaluate, create, and communicate information using digital technologies—is widely recognized as a critical 21st-century competence (Tinmaz et al., 2022; Peng and Yu, 2022). Beyond technical proficiency, it encompasses critical thinking, ethical participation, digital citizenship, and self-regulated learning (Law et al., 2018). As universities intensify their focus on preparing graduates for the digital economy, digital literacy has become foundational not only for academic success but also for effective participation in technology-mediated workplaces.

Career competence—students’ perceived readiness and capability to transition into employment—has likewise gained prominence in the digital era (Jo et al., 2024; Presti et al., 2022). Employers increasingly expect adaptability, digital fluency, and entrepreneurial mindsets developed across formal and informal learning channels. Social media sits at this intersection: it provides spaces for informal learning, professional branding, portfolio building, and network formation that can support career exploration and opportunity seeking.

At the same time, research in Asian contexts often emphasizes risks (e.g., distraction, addiction) more than developmental benefits (Mohamad et al., 2023). Many studies also rely primarily on cross-sectional surveys and underutilize theory to explain how motivations and behaviors shape digital engagement and outcomes (Alshalawi, 2022b,2022c). These tendencies constrain understanding of both mechanisms (why and how social media might build competencies) and outcomes (which competencies change and under what conditions).

To address these gaps, this study examines the role of structured social media use in enhancing digital literacy and career competence among university students in Southern China. A convergent parallel mixed-methods design was employed to capture both measurable relationships and lived experiences: quantitative analyses assessed associations among social media use, digital literacy, and career competence, while qualitative semantic techniques illuminated how students describe and make meaning of their learning and professional development online. The study is guided by Social Cognitive Career Theory (Lent et al., 1994), which emphasizes self-efficacy, outcome expectations, and goals in career development, and by gamified learning theory (Deterding et al., 2011), which highlights how engagement and feedback mechanisms embedded in digital platforms can sustain motivation and scaffold skill growth.

Accordingly, this study addresses the overarching question:

How does structured social media use influence university students’ digital literacy and career competence?

To further explore this overarching inquiry, the study is guided by four specific research questions:

1. How does structured use of social media platforms influence university students’ digital literacy?
2. How does structured use of social media platforms influence university students’ career competence?
3. What is the relationship between digital literacy and career competence among university students?
4. What qualitative themes and behavioral patterns characterize students’ social media practices in relation to digital literacy and career competence?

This paper makes three contributions. First, it provides empirical evidence from an Asian context (Southern China), addressing regional imbalances in the digital education literature. Second, it integrates SCCT with gamified learning theory to articulate mechanisms linking social media engagement to skill development, offering a theoretically grounded account of how self-efficacy, outcome expectations, and platform feedback loops operate together. Third, it contributes methodological breadth by combining quantitative modeling with qualitative semantic analysis, yielding a richer, mechanism-focused understanding of students’ digital practices and their implications for employability.

The novelty of this study lies in its theoretical integration of Social Cognitive Career Theory (SCCT) and gamified learning principles. By combining these frameworks, the research explains how social media engagement can simultaneously enhance self-efficacy, motivation, and skill acquisition through feedback-oriented and goal-driven learning experiences. This theoretical synthesis advances understanding of how technology-mediated environments can nurture both digital literacy and career competence in Asian higher education.

The remainder of this paper is structured as follows: Section 2 reviews the literature, Section 3 outlines the theoretical framework, Section 4 describes the methodology, Section 5 presents the results, Section 6 discusses the findings, and concludes with implications and directions for future research.

## 2 Literature review

Digital literacy is a multifaceted construct that encompasses individuals’ ability to effectively navigate, evaluate, and create information using digital technologies. It involves not only technical proficiency but also higher-order cognitive and social competencies that enable users to critically assess information and participate in digital environments responsibly. Recent research by Zhu et al. (2025) highlights that digital literacy develops through situated and interactive experiences in digital contexts, where students’ perception of digital situations significantly shapes their literacy outcomes. Furthermore, peer interaction and self-efficacy play crucial mediating roles in enhancing learning outcomes in digital environments (Pan et al., 2024). Within this framework, career competence can be viewed as an extended dimension of digital competence, encompassing technological, informational, and socio-communicative literacies essential for employability in knowledge-based economies. Social media engagement, as a form of digital participation, reflects individuals’ active interaction and content creation behaviors in online platforms, serving as both a component and facilitator of digital literacy and professional competence development.

### 2.1 Social media and informal learning

Social media has transformed from being primarily leisure-oriented to a powerful medium for informal learning, where knowledge is shared through user-generated content, peer-to-peer interactions, and networked communities. Platforms such as TikTok, WeChat, Facebook, LinkedIn, and Xiaohongshu provide students with opportunities to observe, replicate, and create content while receiving real-time feedback (Yang and Wang, 2021). These affordances align with constructivist learning principles, as Siemens (2005) noted, by enabling autonomy, collaboration, and active meaning-making in digital environments. While Manca and Ranieri (2016) emphasize the risks of social media use, Greenhow et al. (2023) point to its creative potential—indicating an unresolved tension that this study addresses.

From the perspective of Social Cognitive Career Theory (SCCT), informal learning on social media constitutes significant “learning experiences” that enhance students’ self-efficacy and outcome expectations (Al-Hattami, 2025). For example, successfully applying a digital skill acquired via social media can strengthen confidence in managing academic or professional tasks, which in turn shapes career-related goals. At the same time, gamified learning theory provides insight into why such engagements are sustained. Features like likes, shares, comments, and algorithmic visibility operate as feedback loops and rewards systems that encourage iterative participation, reinforcing self-directed learning behaviors.

However, research in Asian contexts remains skewed toward examining the risks of social media use—such as distraction, dependency, or superficial engagement—rather than its capacity to foster agency, creativity, and employability-related skills (Chugh and Ruhi, 2018). As a result, students’ informal digital practices are often marginalized in academic discourse despite their increasing relevance to career development. A more nuanced understanding of how these practices can cultivate transferable skills is therefore essential.

### 2.2 Digital literacy and higher education

Digital literacy is widely recognized as a core graduate attribute in higher education and a vital 21st-century skill. While early definitions focused narrowly on effective use of digital tools, contemporary perspectives emphasize broader competencies, including information evaluation, media literacy, ethical participation, communication, and identity management (Ng, 2012). These competencies are essential not only for academic success but also for employability, where adaptability, critical thinking, and responsible digital citizenship are expected.

International frameworks illustrate both the progress and ongoing challenges in digital literacy development. The UK’s JISC framework identifies seven dimensions of digital capability, including collaboration, identity, and problem-solving (Fazel and Sayaf, 2025), whereas UNESCO positions digital literacy as foundational for lifelong learning and workforce readiness. Nevertheless, disparities persist: Van Laar et al. (2017) note gaps in students’ preparedness due to unequal access to resources and institutional support, while Ramli et al. (2023) argue that in Asia, digital literacy initiatives remain fragmented and insufficiently linked to students’ informal digital practices, particularly on social media. This contrast highlights an unresolved tension between formal educational efforts and students’ lived experiences, a gap this study seeks to address.

Theoretical perspectives help clarify this gap. From an SCCT lens, students’ engagement with digital literacy is influenced by their outcome expectations: those who believe that developing digital skills enhances career opportunities are more likely to persist. Gamified learning theory complements this view by explaining how platform affordances—such as recognition, visibility, and incremental progress (e.g., follower growth, badges, feedback)—sustain motivation to refine digital competencies. Together, these perspectives illustrate that digital literacy development is not purely technical but also motivational and behavioral.

Despite these insights, empirical research remains limited in showing whether informal digital practices, particularly via social media, translate into measurable literacy gains. This disconnect between students’ everyday digital behaviors and formal assessments represents a critical tension in the literature, reinforcing the need for studies that integrate both theoretical and contextual perspectives.

### 2.3 Career competence

Career competence, broadly defined as the readiness and perceived ability to transition into the labor market with confidence and adaptability, is increasingly shaped by digital practices (Jo et al., 2024). In the digital economy, employability now depends on a blend of digital fluency, networking capability, and online identity management. Social media sits at the intersection of these domains: LinkedIn functions as a professional networking platform, while content-sharing spaces such as YouTube or Xiaohongshu allow students to develop portfolios, creative outputs, and personal brands.

SCCT provides a useful explanatory lens here. Students’ active participation in professional communities online represents learning experiences that can shape self-efficacy and outcome expectations, both of which are central to career development. Recognition from peers or professionals on social platforms can enhance confidence in one’s employability, while exposure to role models and professional networks can influence career goals and aspirations (Shen et al., 2024). Gamified mechanisms embedded in these platforms—such as algorithmic visibility, follower counts, and competitive challenges—further reinforce engagement, motivating students to persist in skill development and career exploration.

Empirical studies affirm these connections. Research has shown that social media supports career exploration, self-marketing, and transferable skill acquisition such as collaboration and problem-solving (Cai et al., 2020; Khedher, 2015). Recent contributions extend this view by emphasizing the emergence of digital career competencies—adaptability, digital fluency, and self-directed learning—as critical dimensions of employability (de Villiers Scheepers et al., 2024). Nevertheless, most existing work continues to focus narrowly on networking platforms like LinkedIn, thereby neglecting the broader ecology of social media practices that students engage with daily. Furthermore, according to Chang and Kabilan (2024) academic programs often fail to integrate social media literacy into career development curricula, leaving students underprepared to leverage digital platforms effectively for professional growth.

Taken together, these studies suggest that while social media holds significant potential for developing career competence, its role remains under-theorized and insufficiently embedded in educational practice.

### 2.4 Methodological imbalance (gap)

Despite the growing body of scholarship on social media, digital literacy, and employability, several critical gaps remain. These gaps restrict the field’s ability to provide theoretically grounded, methodologically robust, and regionally inclusive insights into how university students can leverage social media for both learning and career development. The following four gaps are particularly salient.

#### 2.4.1 Gap 1: fragmented constructs

A central limitation in the literature is the fragmentation of key constructs. Much of the existing research treats social media use, digital literacy, and career competence as separate domains of inquiry rather than exploring their interconnections. Studies focusing on social media often emphasize recreational or social functions, with limited attention to transferable competencies such as collaboration, digital problem-solving, or critical thinking (Manca and Ranieri, 2016; Chugh and Ruhi, 2018). Digital literacy studies, meanwhile, tend to privilege technical skills or information evaluation while overlooking their relevance for career development (Ng, 2012; Tinmaz et al., 2022). At the same time, scholarship on career competence foregrounds adaptability and employability skills (Jo et al., 2024) but rarely examines how these skills are cultivated through informal, digitally mediated practices such as professional networking, peer learning, or online self-branding.

This siloed approach restricts theoretical progress and limits practical application. As Van Laar et al. (2017) argue, 21st-century competencies—including communication, collaboration, and creativity—are inseparable from digital capabilities. Yet without integrated frameworks, existing studies present only partial or isolated perspectives. For example, Chang and Kabilan (2024) highlight how social media can serve as e-portfolios supporting reflection and skill demonstration, but their work remains disconnected from broader digital literacy or employability models. The absence of integrative studies leaves a blind spot: the mechanisms by which students’ informal learning behaviors on social media contribute to both digital literacy and career readiness remain poorly understood. Addressing this fragmentation requires a holistic framework that explains how digital practices translate into meaningful academic and professional outcomes.

This gap indicates that future research should adopt integrative frameworks linking social media use, digital literacy, and career competence to capture the interconnected nature of 21st-century learning.

#### 2.4.2 Gap 2: limited theoretical grounding

A second weakness lies in the limited use of theoretical frameworks. Many studies remain largely descriptive, documenting patterns of social media use or reporting correlations with academic outcomes, without explaining the underlying mechanisms. For instance, Alshalawi (2022a) examines social media adoption in higher education but provides little theoretical interpretation of why intensity of use matters for learning outcomes. Similarly, even large-scale systematic reviews, such as Satapathy et al. (2024) on the burden of gaming disorder among adolescents, synthesize valuable empirical evidence yet fall short of embedding their findings within explanatory models of digital behavior. Such descriptive or epidemiological approaches highlight important social concerns but leave unanswered questions about the mechanisms through which digital engagement shapes learning, wellbeing, or employability.

This absence of explanatory depth hinders cumulative knowledge-building and reduces the transferability of findings across contexts. Scholars have repeatedly noted this shortcoming. Greenhow et al. (2020) argue that inconsistent use of theory weakens the explanatory power of technology-enabled learning research, while Greenhow and Lewin (2021) emphasize the need for robust pedagogical models to make digital learning research more generalizable. Within employability research, the absence of career development theories such as Social Cognitive Career Theory (SCCT) is especially striking. SCCT (Lent et al., 1994) highlights self-efficacy, outcome expectations, and goals as key mechanisms shaping career development, yet few studies use this lens to interpret how social media engagement influences career trajectories. Likewise, gamified learning theory (Deterding et al., 2011), which explains how feedback, rewards, and social interaction sustain engagement, has seldom been applied to understand students’ informal digital practices.

Consequently, future research must move beyond descriptive or correlation-based accounts to explicitly embed social media studies within established theoretical frameworks. Doing so will not only clarify how and why digital engagement fosters literacy, wellbeing, and employability, but also ensure that findings are cumulative, transferable, and relevant to broader scholarly debates.

To address this theoretical fragmentation, the present study integrates Social Cognitive Career Theory (SCCT) and gamified learning theory as complementary lenses. SCCT explains how self-efficacy, outcome expectations, and goals drive students’ academic and career behaviors, whereas gamified learning theory elucidates how digital platforms sustain motivation through feedback and reward mechanisms. Together, they provide a more holistic account of how social media engagement can influence both psychological and behavioral dimensions of learning and career development. This theoretical integration directly responds to calls for deeper explanatory models that move beyond descriptive accounts of digital participation.

This gap indicates that future studies should embed social media research within robust theoretical models—such as SCCT and gamified learning theory—to explain underlying mechanisms of digital engagement.

#### 2.4.3 Gap 3: methodological imbalance

A third limitation is methodological imbalance, particularly the over-reliance on quantitative surveys and regression-based analyses. While such approaches yield breadth and generalizability, they often reduce complex experiences into simplistic metrics. As Jo et al. (2024) argue, career competence is multidimensional and shaped by both subjective self-perceptions and observable behaviors, which cannot be fully captured through questionnaires alone. Recent scholarship highlights the need for greater methodological diversity. For example, Hoi and Hang (2021) employed a mixed-methods design to examine students’ use of Facebook for learning, revealing motivational and contextual factors that would have been overlooked in purely quantitative studies. Similarly, Phutela and Dwivedi (2020) explored students’ adoption of e-learning through qualitative inquiry, uncovering cultural and affective influences that structured surveys could not fully capture. Other research has demonstrated the utility of qualitative and mixed approaches in digital education, such as Ghanbarzadeh and Ghapanchi’s (2023) qualitative study on drivers of student engagement in 3D educational spaces and Arslantas and Gul’s (2022) mixed-methods analysis of digital literacy among students with visual impairments. Collectively, these works illustrate that while qualitative and mixed-methods research can provide deeper contextual and experiential insights, they remain relatively underutilized compared to survey-based designs. This imbalance restricts the ability of scholarship to capture the full complexity of digital engagement and its consequences. Accordingly, more interpretive and integrative methodologies—such as interviews, text analysis, or semantic network mapping—are needed to complement quantitative surveys, enrich theoretical insight, and better inform institutional practice.

This gap highlights the need for mixed-methods and qualitative approaches to capture the nuanced and contextual dimensions of students’ digital learning experiences.

#### 2.4.4 Gap 4: regional underrepresentation

Finally, there is a pronounced regional imbalance in the literature. As noted by Shibambu and Mojapelo (2024), research on social media, digital literacy, and employability remains dominated by Western contexts, particularly North America and Europe, while studies from Southeast Asia and China are limited (Simanjuntak et al., 2025). This underrepresentation restricts both the inclusivity and global applicability of current models.

For instance, Mohamad et al. (2023) reviewed social media for English learning in Southeast Asia and highlighted substantial cultural and pedagogical differences from Western contexts. Similarly, Farley and Song (2019) documented unique infrastructural and linguistic barriers in Southeast Asian mobile learning. Such findings underscore that Western results cannot be directly generalized to Asian higher education. Recent efforts—such as Ramli et al. (2023) on digital literacy in Malaysia and China, and Tee et al. (2024) on Malaysian employers’ views of graduate skills—begin to address this imbalance, but contributions remain sporadic.

The implications are twofold. Theoretically, models developed primarily in the West risk ignoring the affordances and challenges of Asian digital ecosystems. Practically, educators and policymakers in the region lack locally grounded evidence to inform interventions. Closing this gap requires more context-sensitive, empirically robust research situated within non-Western higher education systems.

This gap underscores the necessity for regionally grounded research in non-Western higher education systems to develop culturally relevant models of digital literacy and employability.

Taken together, these four gaps highlight the fragmented, under-theorized, and regionally imbalanced state of current research at the intersection of social media, digital literacy, and career competence. To advance the field, this study proposes an integrated framework combining SCCT and gamified learning theory, employs a mixed-methods design to capture both quantitative patterns and qualitative insights, and situates the analysis in a non-Western context to enrich global perspectives on students’ digital and professional development.

### 2.5 Research hypotheses

The formulation of research hypotheses is central to establishing the logical bridge between theory and empirical investigation. In this study, hypotheses are developed based on Social Cognitive Career Theory (SCCT) (Lent et al., 1994) and gamified learning theory (Deterding et al., 2011). SCCT emphasizes the roles of self-efficacy, outcome expectations, and goals in shaping career-related behaviors, while gamified learning theory explains how interactive and feedback-rich digital environments foster sustained motivation and skill development. Together, these frameworks provide complementary perspectives for understanding how social media engagement can influence students’ digital literacy and career competence. In addition, prior empirical research on digital skills, social media use, and employability offers evidence to support the hypothesized relationships. The following subsections elaborate on the theoretical and empirical rationale for each hypothesis.

#### 2.5.1 Social media and digital literacy

From the perspective of SCCT, social media can strengthen students’ self-efficacy in navigating digital environments. Frequent engagement with features such as information search, content creation, and peer interaction builds confidence in digital tool use and fosters continuous learning (Tang et al., 2008). Outcome expectations also play a role: students who believe that digital participation will enhance their knowledge and future opportunities are more likely to persist in digital learning behaviors.

Gamified learning theory further supports this connection, as social media platforms integrate features like likes, shares, follower counts, and algorithmic visibility, which function as feedback mechanisms that reinforce learning behaviors (Deterding et al., 2011). These mechanisms encourage students to refine their digital communication, critical evaluation, and information-sharing practices, all of which align with the multidimensional nature of digital literacy (Tinmaz et al., 2022).

Empirical studies provide converging support. Manca and Ranieri (2016) found that Facebook and other platforms promote informal skill development, while Chugh and Ruhi (2018) highlighted the role of social media in fostering self-regulated learning. Van Laar et al. (2017) systematically linked digital skills with broader 21st-century competencies, suggesting that social media can be a conduit for acquiring these abilities. Thus, it is hypothesized:

***H1: Structured use of social media platforms significantly improves university students’ digital literacy.***

#### 2.5.2 Social media and career competence

Social Cognitive Career Theory emphasizes that students’ outcome expectations—beliefs about how present behaviors influence future opportunities—play a pivotal role in career development (Lent et al., 1996). When students perceive that social media enhances professional visibility, networking, or employability, they are more likely to engage strategically with these platforms. According to Jo et al. (2024), social media can also shape career-related self-efficacy, as students gain confidence in exploring opportunities, showcasing skills, and connecting with professionals.

From a gamified learning perspective, social media’s reward mechanisms and visibility cues act as motivational reinforcers for career-oriented engagement. For example, posting a portfolio on Xiaohongshu or LinkedIn and receiving feedback can encourage persistence in self-marketing, identity building, and reflective learning. These iterative experiences resemble gamified progress cycles that reinforce career readiness behaviors.

Empirical evidence supports this reasoning. Cai et al. (2020) demonstrated that social media facilitates career exploration and skill-building, while Khedher (2015) highlighted its role in personal branding. More recently, Chang and Kabilan (2024) showed that social media-based e-portfolios promote career identity formation and skill demonstration. Collectively, these findings suggest that purposeful engagement with social media enhances career competence.

***H2: Structured use of social media platforms significantly enhances university students’ career competence.***

#### 2.5.3 Digital literacy and career competence

Digital literacy has been increasingly recognized as a foundation for employability, enabling graduates to adapt to rapidly evolving workplace demands. Within SCCT, digital literacy can be interpreted as both a form of self-efficacy (confidence in using digital tools) and a critical resource shaping outcome expectation regarding career success. Students with higher digital literacy are better positioned to leverage online opportunities, navigate digital workflows, and pursue career goals with greater confidence.

Gamified learning theory complements this view by suggesting that the incremental mastery of digital tasks fosters a sense of progress and accomplishment, reinforcing persistence in career-oriented learning. For instance, students who successfully manage digital collaboration tools or engage in online content creation not only gain technical competence but also develop the adaptive learning habits necessary for career readiness.

Empirical studies reinforce this linkage. Van Laar et al. (2017) connected digital literacy with essential 21st-century skills such as problem-solving and communication, both of which are valued in the labor market. Presti et al. (2022) emphasized the importance of career adaptability and digital fluency for employability. Tee et al. (2024) provided Southeast Asian evidence that employers demand graduates with strong digital skills, directly tying digital literacy to employability outcomes. Therefore, it is hypothesized:

***H3: Digital literacy positively predicts career competence among university students.***

#### 2.5.4 Qualitative insights into social media, digital literacy, and career competence

While quantitative models can capture relationships between variables, SCCT and gamified learning theory suggest that individual experiences and contextual factors also shape digital and career development outcomes. Students’ narratives reveal how they interpret, regulate, and sustain their engagement with social media for learning and employability. For example, self-regulation, professional identity formation, and cognitive reframing of social media from entertainment to learning are qualitative dimensions that complement quantitative findings.

Mixed-methods approaches are particularly valuable in this regard. Manca and Ranieri (2016) argued that understanding social media learning requires capturing students’ subjective experiences, while Greenhow et al. (2023) emphasized that teacher and learner perspectives provide unique insights often missed in survey-based research. By combining statistical patterns with thematic analysis, this study seeks to build a more comprehensive account of how social media influences students’ development.

Accordingly, the fourth hypothesis reflects the integrative role of qualitative exploration in complementing quantitative findings:

***H4: Students’ qualitative experiences of social media use (e.g., self-regulation, employability focus, cognitive reframing) complement and explain the quantitative improvements observed in digital literacy and career competence.***

Collectively, these strands of research suggest a sequential relationship in which structured social media engagement fosters digital literacy, which in turn enhances students’ career competence. Social media platforms offer informal yet purposeful learning spaces that develop technical and cognitive digital skills; these skills subsequently translate into employability outcomes such as adaptability, self-efficacy, and professional visibility.

Building upon these conceptual linkages and addressing the fragmented approaches observed in prior studies, the following section presents the study’s dual-theory framework, which integrates Social Cognitive Career Theory and gamified learning theory. This integrated lens provides a coherent explanation of how structured social media use shapes students’ digital literacy and career competence.

## 3 Theoretical framework

### 3.1 Social Cognitive Career Theory

Social Cognitive Career Theory (SCCT), first articulated by Lent et al. (1994) and grounded in Bandura’s (1999) Social Cognitive Theory, has become one of the most influential frameworks in career development research. SCCT emphasizes that career-related behaviors emerge from the reciprocal interplay among personal factors (e.g., beliefs, interests, and affect), behavioral factors (e.g., skill acquisition and practice), and environmental factors (e.g., opportunities, supports, and barriers). Central to the model are three constructs: self-efficacy, outcome expectations, and goals. Together, these constructs explain how individuals develop career interests, form choices, and pursue career achievements.

Self-efficacy refers to an individual’s belief in their capacity to successfully execute a specific task. Within the context of higher education, self-efficacy strongly predicts students’ willingness to experiment with new technologies, develop digital competencies, and pursue online learning opportunities (Cosby et al., 2023). For instance, students who feel confident in their ability to navigate social media platforms for professional networking are more likely to invest effort in building online portfolios and engaging with potential employers (Jeon and Kim, 2022). Outcome expectations concern beliefs about the likely consequences of performing certain actions. In the digital era, outcome expectations include students’ perceptions that purposeful engagement with social media will result in enhanced employability, improved digital literacy, and expanded career opportunities (Lent, 2002). Finally, goals represent the intentions and commitments to undertake particular actions, such as consistently curating a professional LinkedIn profile or engaging in online collaborations that align with long-term career aspirations.

Applied to this study, SCCT provides a lens through which to examine how university students’ social media use influences their academic and career trajectories. Specifically, social media engagement (personal factor) interacts with behavioral practices such as digital skill-building, while also being shaped by environmental supports (institutional encouragement, digital infrastructure, peer modeling). For example, when students observe peers successfully leveraging platforms such as LinkedIn, Xiaohongshu, or Bilibili for internships or skill promotion, they may develop stronger self-efficacy beliefs and outcome expectations, reinforcing their own digital career exploration. Thus, social media becomes both a site of informal learning and a mechanism for career self-management, in line with SCCT’s assumptions (Lent and Brown, 2013).

A growing body of research demonstrates SCCT’s applicability in digital and employability contexts. For instance, Cosby et al. (2023) showed that digital literacy among university students was strongly mediated by self-efficacy, underscoring the importance of confidence in digital environments. Similarly, Jo et al. (2024) emphasized that career competencies—such as adaptability, networking, and self-promotion—can be effectively explained through SCCT constructs, particularly self-efficacy and outcome expectations. Wang et al. (2022) extended this perspective by arguing that SCCT is especially valuable in the digital era, as it helps illuminate how students from diverse backgrounds use online resources to access career development opportunities. Together, these findings highlight SCCT’s relevance not only in traditional career counseling but also in understanding how digital environments, including social media, foster employability.

Moreover, SCCT’s focus on environmental affordances is especially pertinent in Asian higher education contexts, where institutional structures and cultural values shape students’ engagement with technology. For example, Tee et al. (2024) reported that Malaysian employers increasingly demand digital skills and career adaptability, yet many graduates demonstrate gaps in preparedness. From an SCCT perspective, this skills gap may reflect inadequate environmental supports (e.g., insufficient integration of social media literacy into curricula) and limited exposure to positive role models who demonstrate professional digital practices. By situating this study in Southeast Asia, where empirical research remains scarce, the framework helps explain how structural conditions interact with personal beliefs and goals in shaping digital literacy and career competence.

Importantly, SCCT also offers a developmental and motivational explanation for students’ career preparation behaviors. Lent and Brown (2013) expanded SCCT into the social cognitive model of career self-management, which emphasizes proactive strategies such as networking, self-promotion, and digital identity construction. In the context of this study, students’ ability to strategically engage with social media can be seen as a form of career self-management: those with stronger digital self-efficacy are more likely to explore online learning opportunities, those with positive outcome expectations are more inclined to invest time in professional networking, and those with clear career goals are better positioned to transform everyday social media use into purposeful employability practices.

While SCCT has been extensively validated in Western settings, its application in non-Western digital contexts is still limited. Recent studies underscore the importance of extending SCCT into emerging career development challenges, including the integration of informal digital practices and gamified learning environments (Wang et al., 2022). This study therefore extends SCCT in two ways: first, by situating it within students’ informal digital practices (such as everyday social media use) rather than formal academic settings; and second, by examining its applicability to career competence development in Southeast Asian higher education. In doing so, the study responds to calls for more context-sensitive applications of SCCT that account for cultural and technological diversity.

In sum, SCCT offers a robust theoretical foundation for this study by clarifying how social media engagement influences students’ digital literacy and career competence through the interconnected pathways of self-efficacy, outcome expectations, and goals. By framing social media use as both a personal and environmental affordance, the theory highlights how students’ beliefs, motivations, and behaviors converge to shape their readiness for the digital labor market. This study contributes to the growing body of scholarship that extends SCCT into digital and cross-cultural contexts, thereby enriching our understanding of career development in the 21st century.

### 3.2 Gamified learning theory

Gamified learning theory provides a complementary lens to Social Cognitive Career Theory (SCCT) by explaining how specific features of digital environments foster sustained engagement, motivation, and skill development. Gamification broadly refers to the use of game-like design elements—such as points, badges, leaderboards, levels, and feedback systems—in non-game contexts to influence behaviors and enhance participation (Deterding et al., 2011). Unlike traditional instruction, which often relies on extrinsic assessment structures, gamified approaches integrate motivational design into everyday activities, making learning processes more engaging, interactive, and rewarding.

A large body of scholarship has examined the educational potential of gamification. For instance, Sailer and Homner (2020) conducted a meta-analysis and found that gamification positively influences both cognitive and motivational learning outcomes, particularly when combined with feedback mechanisms and meaningful goal structures. Similarly, Zaric et al. (2021) argue that the effectiveness of gamified learning depends on the learner’s own tendencies and learning styles; while some individuals thrive in competitive or rewards-driven environments, others benefit more from collaborative or mastery-oriented features. These insights highlight that gamification should not be treated as a one-size-fits-all mechanism but rather as a nuanced pedagogical tool that can be adapted to diverse learner needs.

Within the context of social media, gamification is not an external add-on but a built-in structural feature. Platforms such as TikTok, WeChat, Xiaohongshu, and LinkedIn naturally embed gamified elements such as follower counts, likes, algorithm-driven visibility, and streaks or participation challenges. These features shape user engagement by offering immediate feedback, recognition, and visible markers of achievement. Over time, they reinforce patterns of voluntary and sustained participation, mirroring principles of gamified learning (Kulkarni et al., 2022). For university students, such mechanisms can transform casual online activity into meaningful opportunities for developing digital literacy, as they learn how to communicate effectively, curate content strategically, and present themselves professionally to wider audiences.

Gamified learning theory also emphasizes progression and mastery, which are particularly relevant in digital literacy development. For example, students who create content regularly—such as tutorial videos or reflective blogs—often track improvements in audience engagement metrics, which serve as proxies for skill mastery (Chan et al., 2024). In this way, gamified features contribute to building students’ self-regulated learning capacities, reinforcing SCCT’s notion of self-efficacy. Students not only gain confidence in their digital abilities but also see tangible outcomes from their efforts, which further motivates them to pursue more ambitious goals in both academic and career domains.

Crucially, gamified mechanisms extend beyond academic engagement to the sphere of career development. Professional networking platforms like LinkedIn employ explicit gamified cues, including profile strength indicators, endorsement counts, and skill badges, which encourage students to actively build and display career competencies. Recent scholarship has argued that these gamified career tools can enhance employability by fostering reflection, encouraging proactive career behaviors, and increasing professional visibility (Leeming and Rossier, 2025). This aligns with the broader argument that gamified learning environments contribute not only to knowledge acquisition but also to sustainable wellbeing and career preparedness in the digital age (Lee, 2023).

Nevertheless, it is important to acknowledge the limitations of gamification. Some research has cautioned that overemphasis on external rewards, such as likes or badges, may undermine intrinsic motivation and foster superficial engagement (Sailer and Homner, 2020). Moreover, gamification can exacerbate social comparison and digital fatigue if learners feel pressured to constantly perform (Chen and Chang, 2020). Thus, gamification must be strategically integrated, with attention to balancing extrinsic incentives with intrinsic goals of learning and growth. By recognizing these nuances, the current study avoids an overly deterministic view of gamification and instead situates it as one mechanism—among others—that supports motivation and sustained engagement.

In sum, gamified learning theory enriches this study’s conceptual grounding by explaining how digital environments, particularly social media, can transform everyday online activity into iterative learning processes that reinforce self-efficacy, outcome expectations, and goal-directed behavior. While SCCT provides a cognitive–motivational explanation of why students engage in career development activities, gamified learning theory accounts for how the structural and psychological features of social media sustain those activities over time. Taken together, these theories form a dual-pathway framework for understanding the relationship between social media use, digital literacy, and career competence in higher education.

While SCCT and gamified learning theory originate from distinct traditions—cognitive–motivational and design-based perspectives, respectively—their integration provides a unified explanation of how learning and career behaviors unfold in digital contexts. SCCT clarifies why students engage in career-oriented activities on social media through constructs such as self-efficacy, outcome expectations, and goals. Gamified learning theory explains how these motivational tendencies are sustained by environmental mechanisms such as feedback, rewards systems, and progressive challenges. These gamified affordances reinforce self-efficacy by providing immediate feedback, validate outcome expectations through visible recognition, and strengthen goal persistence through incremental progression. Thus, feedback and reinforcement act as connecting mechanisms that translate cognitive motivation into sustained engagement, linking SCCT’s personal factors with gamified learning’s environmental dynamics.

### 3.3 Conceptual framework

This study integrates Social Cognitive Career Theory (SCCT) and Gamified Learning Theory to propose a dual-pathway framework that explains how structured social media use influences students’ digital literacy and career competence. While SCCT emphasizes the cognitive–motivational processes that underlie career-related learning, gamified learning theory highlights the structural and behavioral mechanisms that sustain engagement in digital environments. Taken together, these perspectives provide a comprehensive account of how social media functions as both a learning space and a motivational system for employability preparation.

First, SCCT explains how personal beliefs (self-efficacy), cognitive appraisals (outcome expectations), and intentional behaviors (goals) drive students’ willingness to use social media for skill-building. Within this framework, RQ1 examines whether purposeful engagement with social media enhances students’ digital literacy. The expectation is that as students gain confidence in navigating digital tools and anticipate positive academic or career outcomes, they are more likely to engage in reflective, self-regulated learning that strengthens their digital competencies. Gamified features such as likes, shares, and recognition loops amplify this process by providing immediate feedback that reinforces self-efficacy and sustains digital practice.

Second, SCCT posits that outcome expectations—beliefs about how current actions influence future opportunities—are central to career development. Accordingly, RQ2 addresses whether structured social media engagement improves career competence. Students who perceive professional networking platforms as gateways to internships, employment, or personal branding are more likely to translate digital practices into employability skills. Here, gamification strengthens outcome expectations by providing visible markers of professional progress (e.g., profile strength indicators, endorsement counts, or audience reach), which validate students’ career-related behaviors and encourage persistence.

Third, digital literacy is theorized as a mediating mechanism that connects social media use with career competence. RQ3 explores this relationship by asking whether students’ mastery of digital skills predicts their readiness for the labor market. From an SCCT perspective, digital literacy enhances self-efficacy and reinforces the expectation that technological competence leads to better employment outcomes. Gamified learning theory complements this by showing how the incremental mastery of digital tasks—supported by feedback loops and visible progression—creates a sense of achievement that translates into career adaptability and confidence.

Finally, RQ4 addresses the qualitative dimensions of social media engagement, which capture the lived experiences that cannot be reduced to quantitative indicators. Gamified mechanisms shape students’ affective and behavioral orientations, influencing how they reframe social media from leisure to professional use, develop self-regulation strategies, and internalize digital identities. SCCT provides the cognitive scaffolding for these experiences, while gamification explains the environmental triggers that make sustained engagement possible. Together, these perspectives reveal how individual narratives complement statistical relationships by exposing the subtle interplay of beliefs, motivations, and feedback-driven practices.

By situating this integrated framework in the context of Asian higher education—where rapid digital adoption coexists with uneven institutional support—the study addresses a critical gap in existing scholarship. Previous research has largely treated SCCT and gamification as separate lenses and has focused on Western contexts where digital literacy programs are more systematically embedded in curricula. In contrast, this framework highlights how students in Southeast Asia strategically leverage social media platforms, often informally, to compensate for institutional gaps in career preparation.

**FIGURE 1:**
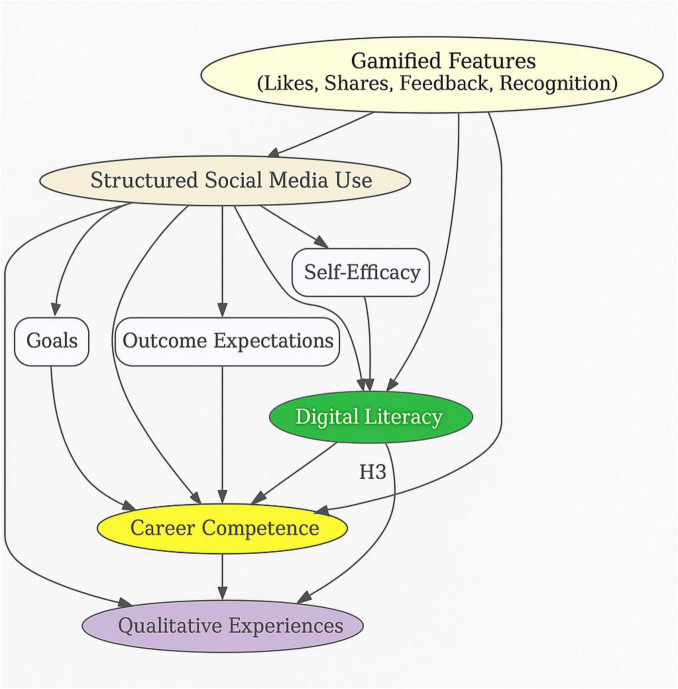
Conceptual framework.

In summary, the conceptual framework in Figure 1 positions social media as both a cognitive–motivational environment (SCCT) and a behavioral–engagement system (gamified learning theory). By integrating these perspectives, the study explains not only whether social media improves digital literacy and career competence, but also how and why these processes unfold in the lived realities of university students.

The integration of SCCT and gamified learning theory also guided the study’s empirical design. Quantitatively, it informed the selection of variables—self-efficacy, digital literacy, and career competence—and the hypothesized mediating relationship among them. Qualitatively, it shaped the exploration of students’ lived experiences with gamified social media use, focusing on motivational reinforcement, feedback cycles, and digital self-regulation. Methodologically, this dual-theory foundation justified the use of a convergent mixed-methods approach, enabling both the measurement of theoretical constructs and the interpretation of behavioral narratives that enrich the quantitative findings.

## 4 Materials and methods

### 4.1 Research design

This study employed a convergent parallel mixed-methods design (Creswell and Plano Clark, 2023) to investigate the impact of university students’ social media engagement on their digital literacy and perceived career competence. This design was selected to collect and analyze quantitative and qualitative data simultaneously, enabling a more comprehensive understanding of the research problem. The integration of both data types provides richer insights than either approach alone.

The convergent parallel mixed-methods design was selected because the research problem required both a broad quantitative understanding of statistical relationships and a deep qualitative exploration of contextual meanings. Unlike sequential models, which prioritize one phase over another, the parallel design allows both strands to be collected and analyzed independently yet with equal priority. This simultaneity ensures that insights derived from statistical associations are directly complemented by participants’ lived experiences without temporal or interpretive lag. Moreover, the topic—students’ social media engagement, digital literacy, and career competence—represents an inherently dynamic and socially embedded phenomenon that benefits from examining cognitive, behavioral, and affective dimensions concurrently.

The rationale for adopting a mixed-methods approach is threefold. First, the quantitative strand identifies the strength and direction of relationships among key constructs (social media engagement, digital literacy, and career competence). Second, the qualitative strand captures students’ lived experiences and the contextualized meanings they assign to these relationships, which cannot be fully captured by numerical measures. Third, integrating the two strands enables triangulation and complementarity, allowing the study to validate statistical patterns while also uncovering the mechanisms and nuances behind them. In this way, quantitative analysis addresses the question of what relationships exist, while qualitative exploration explains how students perceive and enact them.

The study was conducted in two distinct but concurrent phases:

Phase I (Quantitative): Structured questionnaires were administered to assess students’ social media usage patterns, digital literacy levels, and perceived career competence.

Phase II (Qualitative): Open-ended responses were collected to explore students’ personal experiences and perceptions regarding the role of social media in their learning and career development.

Integration occurred at two levels. First, during the interpretation phase, quantitative and qualitative findings were compared and merged through triangulation to identify convergences, divergences, and complementarity. Second, integration was conceptual: qualitative themes were used to explain and enrich quantitative relationships, particularly the mediating role of digital literacy and the mechanisms underlying social media engagement. This joint interpretation strengthened the validity of the conclusions and provided a more holistic understanding of the phenomenon.

### 4.2 Participants and setting

A purposive sampling strategy was employed to recruit 44 undergraduate students from three universities located in southern China. Participants were invited through official university communication channels and class forums, with faculty members assisting in identifying eligible individuals who met the inclusion criteria. This purposive approach ensured variation in academic background and gender balance, thereby reducing potential volunteer bias. Of the 56 invitations distributed, 44 students consented to participate, yielding a response rate of 78%. This sample size is considered suitable for exploratory mixed-methods research that prioritizes data complementarity and interpretive depth rather than statistical generalization.

The inclusion criteria required participants to:

- Be enrolled as full-time undergraduate students;
- Be regular users of at least one social media platform for non-entertainment purposes (e.g., learning, networking, or professional development).

The final sample consisted of an equal gender distribution (22 males and 22 females) with an average age of 21.6 years. Participants represented a range of academic disciplines, including business, education, information technology, and communication. Ethical approval was obtained from the respective university ethics committees, and all participants provided informed consent prior to data collection.

The sample size was deemed adequate for the study objectives. For the quantitative component, statistical power was attainable even with a modest sample, as the analysis primarily examined correlations among continuous variables (Creswell and Plano Clark, 2023). For the qualitative component, thematic saturation was reached after 36 valid open-ended responses, ensuring that no new concepts emerged from additional data. Collectively, these complementary data sources provided sufficient analytical depth and methodological balance to support robust interpretation of the findings.

### 4.3 Ethical considerations

Ethical considerations were embedded throughout the research process. Prior to data collection, ethical clearance was obtained from the participating universities. In line with institutional protocols, all participants were informed about the study’s objectives, their voluntary participation, and their right to withdraw at any time. Written consent was obtained electronically. To ensure confidentiality, no personal identifiers were collected, and data were stored securely with access restricted to the research team. These ethical safeguards were integral to maintaining trust and authenticity in both quantitative and qualitative phases, particularly given the personal nature of participants’ reflections on digital practices.

### 4.4 Instruments and measures

The quantitative survey consisted of three validated scales:

1. Social Media Engagement Scale [adapted from Chugh and Ruhi (2018)], measuring frequency, purpose, and type of use across platforms such as TikTok, Xiaohongshu, and WeChat;
2. Digital Literacy Scale [based on Ng (2012)], assessing students’ self-perceived ability to locate, evaluate, and apply digital information effectively;
3. Career Competence Scale [adapted from Cai et al. (2020)], measuring self-confidence, goal clarity, communication skills, and career exploration behaviors.

All items were rated on a five-point Likert scale (1 = strongly disagree to 5 = strongly agree). Reliability testing showed Cronbach’s alpha values above 0.85 for all subscales, confirming strong internal consistency. Validity was established through expert review and a pilot test.

All survey instruments were originally developed in English and then adapted into Chinese following a rigorous translation and back-translation procedure. Two bilingual experts first translated the items into Chinese, after which an independent translator back translated them into English to check for conceptual equivalence. Minor discrepancies were discussed and resolved through consensus to ensure linguistic and cultural accuracy.

### 4.5 Quantitative data collection and analysis

Data were collected through a self-administered survey distributed across the three participating universities. Participation was voluntary, and responses were recorded anonymously.

The analysis followed a three-step process. First, descriptive statistics were computed to summarize demographic characteristics and central tendencies of the key constructs. Second, Pearson correlation analysis was conducted to examine associations among social media engagement, digital literacy, and career competence. Finally, multiple regression analysis was performed to determine the predictive effects of social media engagement on digital literacy and career competence. Results indicated statistically significant positive relationships among the constructs (*p* < 0.01), with digital literacy serving as a partial mediator between engagement and career competence.

This combination of descriptive, correlational, and regression analyses provided a comprehensive overview of the patterns linking social media use to students’ digital literacy and career competence. These findings informed and were further contextualized by the qualitative strand.

To minimize potential common method bias due to self-reported data, several procedural safeguards were implemented. Respondents were assured of full anonymity and confidentiality to reduce evaluation apprehension. Items were presented in random order, and varied Likert scale anchors were used across constructs to mitigate pattern bias. In addition, data collection was conducted in separate sessions to further minimize the risk of common method variance.

Correlation coefficients among the key constructs were examined to evaluate bivariate relationships and assess potential multicollinearity. Discriminant validity was further confirmed using the Fornell–Larcker criterion, which requires that the square root of the Average Variance Extracted (AVE) of each construct exceed its correlations with other constructs. The results (see Table 1) indicate satisfactory discriminant validity among all variables. As shown in Table 1, the square roots of AVE values for each construct exceeded their inter-construct correlations, indicating satisfactory discriminant validity according to the Fornell–Larcker criterion.

**TABLE 1:** Correlation matrix and discriminant validity.

Construct	1	2	3	4	AVE	√AVE
Social media use	0.82	–	–	–	0.67	0.82
Digital literacy	0.41	0.79	–	–	0.62	0.79
Career competence	0.38	0.46	0.85	–	0.72	0.85

In the convergent parallel mixed-methods design, quantitative and qualitative data were analyzed independently and then integrated during the interpretation phase. Convergence and divergence between findings from both strands were compared to ensure comprehensive understanding and validation of the results.

### 4.6 Qualitative data collection and analysis

In Phase II, participants responded in writing to two open-ended questions:

1. Can you describe how social media has influenced your ability to learn or acquire new knowledge?
2. Has social media helped you prepare for your future career in any way? Please explain.

A total of 36 valid responses were collected. Data were pre-processed (cleaning and segmentation) and analyzed using Python text-mining tools. Term Frequency–Inverse Document Frequency (TF-IDF) identified high-importance keywords, and semantic network analysis mapped co-occurrence patterns to reveal central themes.

Thematic analysis was then conducted to interpret responses. Five major clusters emerged:

1. Learning Motivation (intrinsic and extrinsic drivers of social media learning);
2. Self-Regulation and Discipline (time management and behavioral strategies);
3. Career Awareness and Planning (social media as a career exploration tool);
4. Digital Collaboration and Communication (peer and professional interactions);
5. Platform-Specific Learning Behaviors (distinct practices on different platforms).

Themes were validated through manual coding by two researchers, achieving high intercoder reliability (Cohen’s κ = 0.82). The qualitative findings contextualized quantitative results, illustrating how improved digital literacy was linked to self-regulation strategies and how career competence was supported by identity-building practices on specific platforms.

## 5 Results

### 5.1 Reliability and factor structure of the instrument

The psychometric properties of the measurement instrument were rigorously examined to ensure reliability and construct validity. The overall Cronbach’s alpha coefficient was 0.929, demonstrating excellent internal consistency across items. The Kaiser-Meyer-Olkin (KMO) measure of sampling adequacy was 0.872, and Bartlett’s Test of Sphericity was significant (*p* < 0.001), confirming the suitability of the data for factor analysis.

An Exploratory Factor Analysis (EFA) employing Principal Component Analysis (PCA) with varimax rotation revealed four distinct factors accounting for 74.03% of the total variance. Items with low or cross-loadings (la6, la7, ps5) were removed. The final instrument retained 17 items across four theoretically coherent dimensions in Table 2.

**TABLE 2:** Reliability and factor structure of the instrument.

Dimension	Item codes	Factor loadings
Learning attitude (LA)	la1–la5	0.717–0.804
Career competence (CC)	cc1–cc4	0.697–0.862
Digital literacy (DL)	dl1–dl4	0.753–0.831
Professional skills (PS)	ps1–ps4	0.633–0.836

These results confirm both the construct validity of the instrument and its alignment with the digital capability–employability framework, supporting further analyses.

### 5.2 Pre- and post-test comparison

A 1-month intervention using two social media modalities—gaming and livestream-based content—was implemented. Independent-sample *t*-tests indicated no significant baseline difference between groups (*p* = 0.197), permitting combination into a single intervention group.

Paired-sample *t*-tests comparing pre-test (T0) and post-test (T1) scores revealed significant improvements across all four constructs in Table 3.

**TABLE 3:** Pre- and post-test comparison.

Variable	T0 mean	T1 mean	*P*-value
Digital literacy (DL)	5.48	5.85	0.020
Learning attitude (LA)	4.85	5.40	0.010
Professional skills (PS)	4.72	5.41	<0.001
Career competence (CC)	5.19	5.58	0.010

The results indicate that structured social media use significantly enhanced students’ digital literacy, learning orientation, professional skills, and career confidence. Beyond statistical significance, these gains represent meaningful developmental change consistent with Social Cognitive Career Theory (SCCT). The increased scores suggest that students’ self-efficacy and outcome expectations were strengthened through experiential digital learning, promoting a sense of control and purpose in their career preparation. The improvements in learning attitude and professional skills reflect the cognitive reframing process, whereby digital engagement becomes perceived not as distraction but as a productive academic and professional tool. These findings support Hypothesis 1 (H1) and align with SCCT’s proposition that self-efficacy and outcome expectations improve through experiential engagement.

### 5.3 Hierarchical regression analysis

Hierarchical regression was used to test the predictive role of digital literacy (DL) on related constructs at both baseline (T0) and post-test (T1).

a.    Digital Literacy (DL) → Learning Attitude (LA)
  - T0: β = 0.314, *p* = 0.038, *R*^2^ = 0.099;
  - T1: β = 0.334, *p* = 0.027, *R*^2^ = 0.112.
*Hypothesis 2 supported.*
b.    Digital Literacy (DL) → Professional Skills (PS)
  - T0: β = 0.332, *p* = 0.028, *R*^2^ = 0.110;
  - T1: β = 0.697, *p* < 0.001, *R*^2^ = 0.486.
*Hypothesis 3 strongly supported.*
c.    Digital Literacy (DL) → Career Competence (CC)
  - T0: β = 0.558, *p* < 0.001, *R*^2^ = 0.311;
  - T1: β = 0.685, *p* < 0.001, *R*^2^ = 0.469.
*Hypothesis 4 supported.*

The analyses confirm that digital literacy robustly predicts learning attitude, professional skills, and career competence, with substantially stronger predictive power post-intervention. The notable increase in *R*^2^-values from baseline to post-test indicates a reinforcement effect, suggesting that as students’ digital literacy improved, its influence on employability-related competencies became more pronounced. This supports the conceptualization of digital literacy as a mediating mechanism linking social media learning behaviors and clearer career goals. These findings provide strong engagement to career outcomes. From an SCCT standpoint, these findings exemplify how enhanced self-efficacy in digital contexts translates into proactive evidence for Hypotheses 2–4 (H2–H4). From an SCCT perspective, this reflects the strengthening of self-efficacy and goal clarity through structured digital engagement.

### 5.4 TF-IDF keyword analysis (T0–T2)

Qualitative text responses were analyzed using TF-IDF to capture shifts in salient concepts across three timepoints (T0 = pre-test, T1 = post-test, T2 = projection).

T0 (Pre-test): Keywords such as learning, gaming, and e-sport dominated, suggesting that students initially framed social media use as leisure-driven and sometimes conflicting with academic focus.

T1 (Post-test): Keywords shifted to live stream, e-commerce, and digital capability, reflecting a transition toward skill acquisition and applied learning.

T2 (Future Projection): The terms social media and skills emerged as central, indicating that students envisioned social media as a long-term empowerment and employability tool.

These lexical shifts reinforce the quantitative findings by showing a movement from passive to active and career-oriented digital practices.

This linguistic evolution parallels the statistical improvements in digital literacy and career competence, offering qualitative evidence that students internalized a more self-regulated and future-oriented identity. The convergence between quantitative and textual data thus underscores SCCT’s view that learning experiences reshape efficacy beliefs and career self-management through reflective and goal-directed engagement.

### 5.5 Semantic network and modularity analysis

Semantic co-occurrence analysis mapped the structural integration of concepts at each stage in Table 4.

**TABLE 4:** Semantic network and modularity analysis.

Timepoint	Core nodes	Density	Interpretation
T0	learning - gaming	0.18	Fragmented cognition and leisure conflict
T1	live stream, e-commerce, gaming, digital capability	0.24	Dual focus on skills and industry language
T2	social media, skills	↑	Radiating pattern showing integration and utility

This progression illustrates how students moved from fragmented, leisure-centric perceptions to an integrated, career-embedded digital identity, consistent with SCCT’s career self-management model and gamification’s reinforcement loops.

### 5.6 Emotional polarity analysis

Emotional polarity was examined using the VADER sentiment model (Valence Aware Dictionary and sEntiment Reasoner; Hutto and Gilbert, 2014), which assigns a compound score ranging from −1 (most negative) to +1 (most positive), allowing quantitative tracking of sentiment shifts over time.

Emotion Word Distribution:

- T0: Positive = 51%, Negative = 27%, Neutral = 22%;
- T1: Positive = 87.5%, Negative = 4.5%, Neutral = 8%;
- T2: Positive = 91%, Negative = 4.5%, Neutral = 2%.

Mean Emotion Scores:

- T0: *M* = 1.8 (SD = 2.4);
- T1: *M* = 5.2 (SD = 3.1);
- t(86) = 7.89, *p* < 0.001.

Emotional valence significantly improved across timepoints, suggesting that social media, when structured for learning and career purposes, not only enhances skills but also fosters positive psychological outcomes. This resonates with gamified learning theory, where feedback and recognition mechanisms increase motivation and sustain engagement.

From the perspective of gamified learning theory, this rise in positive emotion reflects the motivational reinforcement loop — feedback, recognition, and perceived progress fuel sustained engagement and confidence. Within SCCT, this corresponds to the enhancement of affective self-efficacy, indicating that emotional reinforcement plays a pivotal role in sustaining digital and career-related motivation.

### 5.7 Integrated summary of findings

Table 5 presents a synthesis of the mixed-methods results, demonstrating how quantitative and qualitative strands converged to support the theoretical framework.

**TABLE 5:** Mixed-methods integration summary.

Method/tool	Key result	Theoretical interpretation
Cronbach’s alpha	0.929–high reliability	Strong measurement validity
EFA (PCA)	Four factors extracted; 74.03% variance explained	Validates multi-dimensional employability framework
Paired *t*-tests	All four constructs improved significantly post-intervention	SCCT: enhanced self-efficacy and outcome expectations
Regression	Digital literacy predicts LA, PS, CC with strong R^2^ (up to 0.486)	SCCT: DL as a driver of career self-management
TF-IDF	Shift from “gaming” → “skills” → “career orientation”	Gamification: feedback drives reframing of digital practices
Semantic network	Structural integration from leisure to empowerment	SCCT + gamification convergence
Emotion analysis	Positive valence rose from 51% → 91%	Gamification: recognition enhances motivation and confidence

Together, the quantitative and qualitative findings converge to depict a coherent developmental pathway: structured social media engagement enhances digital literacy, which in turn strengthens self-efficacy, learning orientation, and perceived career competence. The qualitative progression—from casual gaming discourse to professional skill narratives—further explains how this transformation occurs cognitively and emotionally. This integration supports the theoretical alignment of SCCT and gamified learning, where iterative feedback, cognitive reframing, and emotional reinforcement collectively drive career self-management and employability growth.

In sum, the quantitative results demonstrate that digital literacy functions as a central mechanism linking social media engagement to employability outcomes. Meanwhile, the qualitative strand provides explanatory depth, showing how students’ perceptions, narratives, and emotions evolved throughout the intervention. Together, the findings strongly support the proposed hypotheses and reinforce the integration of Social Cognitive Career Theory and gamified learning theory.

## 6 Discussion

This study examined the impact of structured social media use on university students’ digital literacy and career-related competencies. Anchored in Social Cognitive Career Theory (SCCT), gamified learning theory, and the concept of digital capital, a mixed-methods design was employed to capture both cognitive-behavioral changes and affective responses. The findings advance current theoretical discourse by demonstrating how short-term, structured interventions can reframe social media from a leisure-oriented activity into a vehicle for career development within Asian higher education contexts.

The investigation tested four hypotheses derived from the integrated SCCT–gamification–digital capital framework, and the results provided full empirical support for all proposed relationships. Specifically, H1 and H2 confirmed that structured social media engagement significantly enhances both digital literacy and career competence; H3 validated the mediating role of digital literacy in linking structured social media use to career competence; and H4 was supported by qualitative evidence showing substantial cognitive and emotional restructuring among participants. Collectively, these findings substantiate the study’s conceptual model and extend theoretical understanding of how gamified, structured digital engagement can cultivate employability-oriented skills and professional identity.

### 6.1 Digital literacy as the catalyst

Addressing Research Question 1, this study found that structured social media engagement significantly improved university students’ digital literacy.

The results confirmed that structured social media use significantly enhances digital literacy (DL). Beyond simple frequency of use, this study shows that task-driven and career-oriented engagement fosters deeper digital competence. This extends prior research by Komarudin et al. (2024), which often emphasized passive exposure, by offering experimental evidence that structured digital interventions directly build literacy.

Importantly, digital literacy not only improved quantitatively (*p* = 0.020) but also emerged as a central predictor of career competence (β = 0.685, *p* < 0.001) and professional skills (β = 0.697, *p* < 0.001). This substantiates the SCCT pathway where learning experiences and perceived competence drive career behaviors, highlighting DL as a catalytic construct that bridges social media engagement with employability outcomes.

This finding also responds to Research Question 3, demonstrating that enhanced digital literacy strongly predicts higher career competence. This provides empirical confirmation for H1 and H3.

### 6.2 SCCT in action: from digital tools to career identity

In relation to Research Question 2, the results support that structured digital engagement, mediated by self-efficacy, enhances students’ career competence and identity formation.

Findings provide clear support for SCCT’s cognitive-behavioral loop, where self-efficacy and outcome expectations shape career-related action (Lent et al., 1994). The intervention increased digital task self-efficacy and clarified students’ outcome expectations—they began to recognize social media as a pathway for career advancement.

Notably, the semantic network shift from terms such as “gaming” to “skills,” “e-commerce,” and “career” illustrates identity restructuring: students increasingly saw themselves as active participants in digital economies rather than passive consumers. This contributes to SCCT theory by showing how digital environments function as role models and structural enablers, expanding cognitive scripts for career development. Thus, the study not only confirms SCCT but also extends it by embedding digital media contexts as environments where self-efficacy and outcome expectations are amplified through structured engagement.

These findings thus support H2, underscoring SCCT’s predictive relevance in digital learning environments.

### 6.3 Gamification and the power of feedback

The intervention was grounded in gamification principles, leveraging immersive, feedback-rich formats (livestreaming and gaming). Results highlight the immersion–feedback–motivation cycle: students’ discourse shifted from leisure terms toward professional and learning-oriented keywords within a short intervention period.

This aligns with Hamari et al. (2014), Mekler (2015), who argue that gamified environments heighten motivation and learning persistence. Our findings extend this by demonstrating that gamification does not merely increase engagement but also reshapes students’ emotional valence and career orientation. Emotional polarity rose markedly (positive sentiment from 51% at T0 to 91% at T2), suggesting that gamified design helped reframe perceptions of social media from distraction to empowerment.

In theoretical terms, this study integrates gamification with SCCT: feedback mechanisms in gamified environments enhance self-efficacy and outcome expectations, thereby strengthening the SCCT loop. This integration offers a dual-pathway model where gamification operates as a catalyst for career-related self-efficacy.

These results collectively reinforce the findings addressed in Research Questions 2 and 3, showing that gamified and feedback-rich environments not only enhance students’ motivation and engagement but also strengthen the link between digital literacy and career competence. Thus, the motivational mechanisms described here provide behavioral context for the quantitative pathways observed earlier.

### 6.4 Cognitive restructuring and digital capital

To address Research Question 4, qualitative analyses revealed distinct behavioral and cognitive shifts in students’ engagement patterns, reflecting transformation from entertainment-oriented use toward professional learning orientations.

The text-mining trajectory revealed a shift from fragmented, entertainment-focused interpretations to career-centric perspectives. This resonates with Ragnedda’s (2018) theory of digital capital, which posits that the strategic and critical use of digital tools translates into social and economic advantage.

By reframing social media from consumption to career utility, students effectively converted digital engagement into a form of digital capital. This demonstrates that structured interventions can accelerate the conversion of digital literacy into employability-oriented capital, especially in contexts such as China and Malaysia where platform-based economies (e.g., livestreaming, e-commerce) dominate youth employment pathways.

The study therefore bridges SCCT and digital capital theory: self-efficacy-driven behaviors, when reinforced in digital environments, can accumulate into capital that enhances long-term career trajectories.

These qualitative results provide further evidence for H4.

### 6.5 Comparative insights and local innovation

While prior global studies emphasized structured digital education and socio-cognitive pathways (Livingstone, 2019; Lent et al., 1994), few have experimentally tested short-term, gamified, mixed-media interventions in Asian contexts. This research thus contributes theoretical novelty by showing that even low-cost, localized interventions can generate significant cognitive and emotional restructuring.

The Chinese version of the intervention highlighted the integration of gaming and livestream pedagogy in e-commerce disciplines—a practice particularly salient in Southeast Asia’s digital economies. From this, the study proposes a “technology–emotion–cognition” triadic framework, emphasizing that emotional scaffolding (e.g., confidence-building) is as critical as cognitive training.

Comparatively, this suggests that SCCT and gamification theories, originally developed in Western contexts, can be extended and localized to account for the unique trajectories of Asian digital learners. The emotional polarity findings and semantic density shifts indicate that structured digital interventions can help reconcile the entertainment–work dichotomy, producing a blended identity of students as both learners and future professionals.

### 6.6 Limitations and future research directions

Despite the promising findings, several limitations must be acknowledged to ensure a balanced interpretation of this study. At the same time, these limitations open meaningful avenues for advancing research and practice in higher education.

First, the sample composition presents a key constraint. All participants were drawn from a single discipline—e-commerce—which may have influenced both their familiarity with digital platforms and their perceptions of career relevance. This homogeneity potentially inflated the strength of relationships between structured social media use, digital literacy, and career competence. Students from other academic fields such as the humanities, pure sciences, or technical programs may engage with digital platforms differently, shaping distinct outcome expectations. From the perspective of Social Cognitive Career Theory (SCCT), environmental affordances and self-efficacy beliefs are discipline-sensitive; thus, findings cannot be assumed to generalize across fields. Future research should therefore adopt cross-disciplinary or multi-program designs to validate whether the observed pathways (H1–H3) are consistent across academic contexts. Practically, universities could pilot discipline-specific social media interventions—customized to engineering, education, or arts programs—to align digital literacy development with varied employability trajectories.

Second, the single-site design limits the external validity of the results. Conducted within one Southeast Asian university, the findings may not fully represent the influence of institutional or cultural factors on social media engagement. Prior research indicates that national digital policies, institutional strategies, and cultural norms substantially affect students’ perceptions of media-based learning. Within the SCCT framework, such contextual affordances can either facilitate or constrain career self-efficacy and goal setting. Future studies should thus employ multi-site or cross-national designs to examine whether the SCCT–gamification model holds across different educational systems. For practice, comparative institutional benchmarking could help universities tailor interventions to their local technological infrastructures and labor market needs.

Third, the short intervention period—approximately 30 days—limits inferences about long-term impact and behavioral sustainability. While this study demonstrated significant short-term improvements in digital literacy and career competence, it remains uncertain whether these gains endure over longer durations or translate into tangible employability outcomes. SCCT posits that career development is a cumulative process in which efficacy and outcome expectations evolve through repeated reinforcement. Similarly, gamified engagement often depends on novelty and continuous feedback. Future research should therefore employ longitudinal or follow-up designs that track changes across semesters or full academic programs. Practically, embedding sustained gamified interventions—such as e-portfolios, career challenges, or industry-linked digital projects—could reinforce learning persistence and digital competence over time.

Fourth, the reliance on self-reported data introduces the possibility of bias. Although mixed methods were used, the quantitative strand relied on students’ self-assessments of digital literacy and career competence, which are prone to social desirability and recall bias. Consequently, the relationships observed among engagement, digital literacy, and career competence may be somewhat overstated. Future research should incorporate objective indicators such as performance-based digital skill tests, trace data analytics, or employer assessments. For practice, higher education institutions could complement self-report surveys with learning analytics dashboards or structured employer feedback mechanisms, providing a more balanced and data-driven assessment of career readiness.

Finally, although the qualitative strand enriched the analysis, interpretive bias and context specificity remain potential limitations. The identified themes—self-regulation, employability focus, and cognitive reframing—offered valuable explanatory insight for H4, yet these interpretations are shaped by researcher coding decisions and local context. Methodological triangulation through multiple coders, peer debriefing, or cross-institutional qualitative sampling would strengthen future reliability. Moreover, adopting participatory qualitative methods—such as digital storytelling or reflective blogging—could deepen students’ self-awareness while reducing interpretive bias.

In summary, while the findings must be interpreted cautiously given these constraints, the limitations themselves provide clear pathways for theoretical refinement and practical innovation. Future research should extend across disciplines, institutions, and time horizons, integrating both objective performance measures and qualitative meaning-making. For practice and policy, universities can embed structured, gamified, and reflective social media learning into degree curricula and national digital education strategies. By addressing these limitations through comparative, longitudinal, and multi-method approaches, future scholarship can more robustly determine how structured digital engagement cultivates career competence and digital capital across diverse educational and cultural contexts.

### 6.7 Theoretical contributions

This study makes three key theoretical contributions to the fields of career development, digital learning, and social media studies. First, it extends the scope of Social Cognitive Career Theory (SCCT) beyond traditional, long-term counseling contexts to encompass short-term, media-rich interventions. The findings demonstrate that both digital task self-efficacy and outcome expectations can be meaningfully enhanced within a 30-day structured program, suggesting that SCCT can explain not only gradual but also rapid cognitive and motivational shifts in digital learning environments. Second, this study integrates gamification into career development theory, revealing that gamified and livestreamed learning tasks not only sustain engagement but also facilitate professional identity formation. This integration positions gamification as a dual-pathway mechanism linking behavioral engagement with identity construction—an area previously underexplored in career research. Third, the study provides empirical reinforcement for the digital capital framework by showing how structured social media interventions can convert entertainment-oriented media use into digital empowerment and, ultimately, career-relevant identity capital. The triangulation of quantitative findings with semantic and emotional analyses further enhances the robustness of this theoretical advancement, illustrating the multidimensional nature of employability-oriented digital engagement.

### 6.8 Practical implications

This study yields three major practical implications for educators, policymakers, and practitioners in higher education. First, the substantial rise in positive sentiment—from 51% at T0 to 91% at T2—underscores the importance of integrating emotional scaffolding into employability-oriented interventions. Confidence and motivation emerged as key drivers of sustained engagement and learning persistence, suggesting that emotional design should be treated as a core component rather than a supplementary feature of digital pedagogy. Second, the application of semantic analysis tools such as TF–IDF and modularity mapping introduces a data-informed approach for evaluating cognitive reframing and emotional growth. These tools offer educators and researchers novel metrics for monitoring learning trajectories that go beyond conventional performance indicators. Third, by leveraging students’ existing digital practices such as gaming and livestreaming, this study demonstrates that scalable, low-cost, and culturally responsive interventions can be effectively implemented even within resource-constrained institutional contexts, particularly in emerging digital economies.

### 6.9 Synthesis of theoretical and practical contributions

Taken together, the findings of this study bridge theoretical advancement and practical application in the context of employability development. The results demonstrate that structured social media use can concurrently strengthen self-efficacy and outcome expectations as explained by Social Cognitive Career Theory (SCCT), foster motivational immersion and identity formation through gamification, and transform digital participation from entertainment-oriented behaviors to empowerment and career capital, as articulated in the digital capital framework. Collectively, these outcomes illustrate how theoretically grounded interventions can yield measurable psychological, cognitive, and emotional growth within a short period. Furthermore, the notable rise in positive sentiment underscores the often-overlooked affective dimension of employability, highlighting that emotional scaffolding is integral to sustainable skill and identity development. Validated within the Chinese higher education context, these insights also possess cross-cultural relevance and can be adapted to Malaysia and other emerging digital economies seeking scalable, culturally resonant approaches to enhancing youth employability.

### 6.10 Conclusion

In summary, this study addressed four interrelated research questions. RQ1 confirmed that structured social media use enhances digital literacy; RQ2 demonstrated its positive influence on career competence; RQ3 established digital literacy as a key mediating mechanism between social media engagement and career competence; and RQ4 uncovered qualitative patterns that explain how these changes unfold in practice.

The findings reveal that structured social media engagement—when guided by feedback, reflection, and gamification—can transform university students’ media use from passive consumption into active professional development. Behavioral and cognitive shifts such as self-regulation, employability orientation, and cognitive reframing emerged as central drivers of this transformation.

Theoretically, this research extends Social Cognitive Career Theory (SCCT) into short-term, gamified, and media-rich learning contexts. It demonstrates that self-efficacy and outcome expectations—traditionally seen as long-term constructs—can be rapidly cultivated through immersive digital interventions. The study also advances the digital capital framework by showing how entertainment-oriented practices can be reframed as opportunities for career-relevant learning. Methodologically, the integration of quantitative modeling with computational semantic and sentiment analysis introduces a novel approach for capturing both cognitive and emotional dimensions of digital learning.

From a practical standpoint, the study offers actionable insights for universities, educators, and policymakers. Structured, gamified, and feedback-driven digital interventions can be implemented cost-effectively and at scale to build employability-related skills while aligning with students’ everyday media habits. Extending these interventions across disciplines and longer timeframes—particularly when enhanced by AI-driven personalization—may further sustain students’ digital growth and professional identity.

In essence, this study reframes social media from a space of distraction to a catalyst for digital empowerment. By integrating SCCT and gamified learning principles, it establishes a theoretically grounded and practically viable pathway through which structured engagement fosters digital literacy, career competence, and emotional resilience. The central contribution lies in demonstrating that when purposefully designed, social media can evolve into a transformative learning environment that equips students for employability in the digital era.

## Data Availability

The raw data supporting the conclusions of this article will be made available by the authors, without undue reservation.
